# Supplementary figures and images for: Most frequent South Asian haplotypes of ACE2 share identity by descent with East Eurasian populations
Source: PLoS One. 2020 Sep 16;15(9):e0238255. doi: 10.1371/journal.pone.0238255 (PMC7494073; doi:10.1371/journal.pone.0238255)

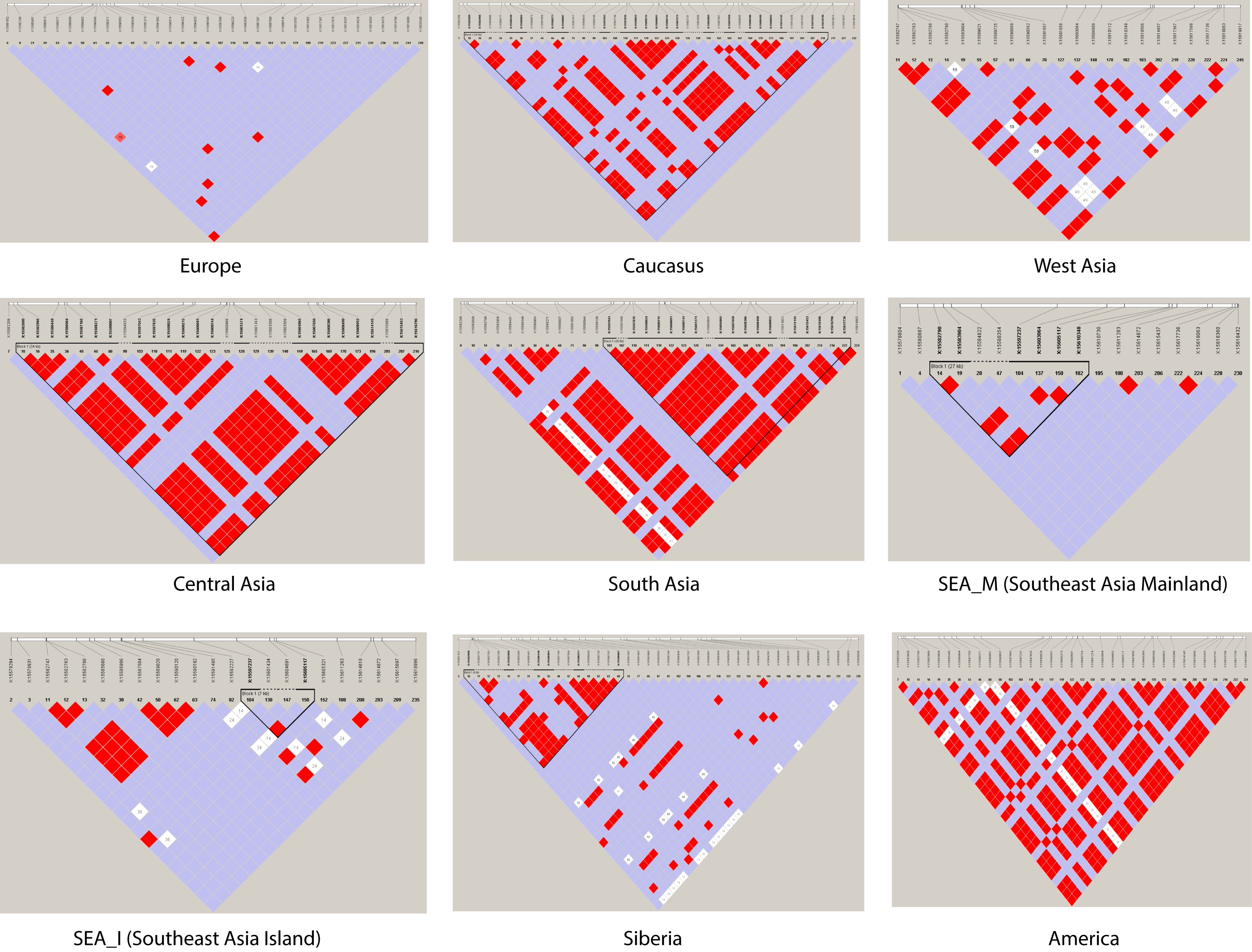

Supplement: S1 Fig — Shading from white to red indicates the intensity of r2 from 0 to 1. Strong LD is represented by a high percentage (>80) and a darker red square. (TIF) [file pone.0238255.s001.tif]

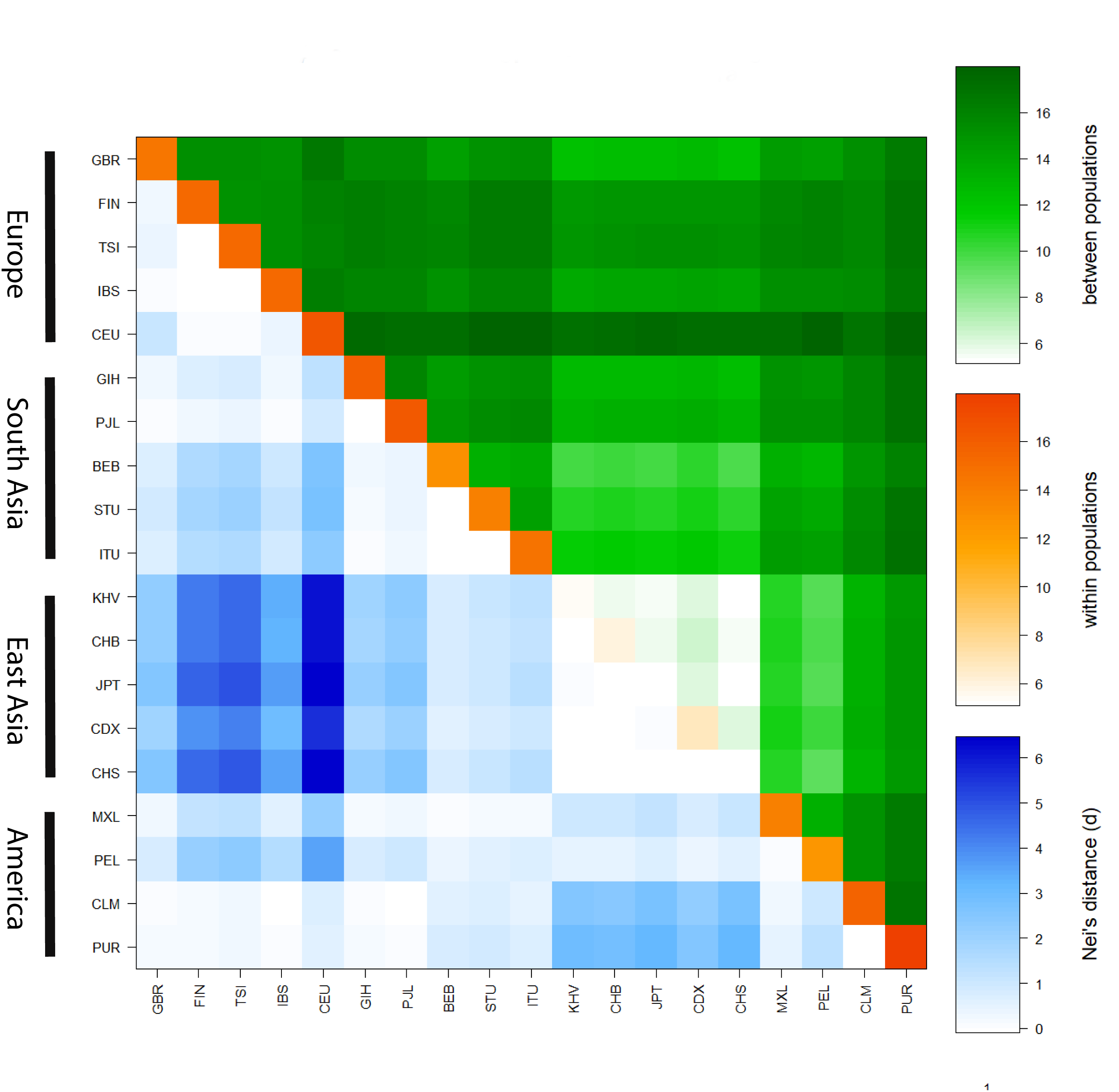

Supplement: S2 Fig — The average pairwise differences between populations are shown in the upper triangle of the matrix (green). The average number of pairwise differences within each population group are shown along the diagonal (orange). The differences between populations based on Nei's genetic distances are depicted in lower triangle of the matrix (blue). The populations are grouped in to superpopulations e.g. European, South Asian, East Asian and American. (TIF) [file pone.0238255.s002.tif]

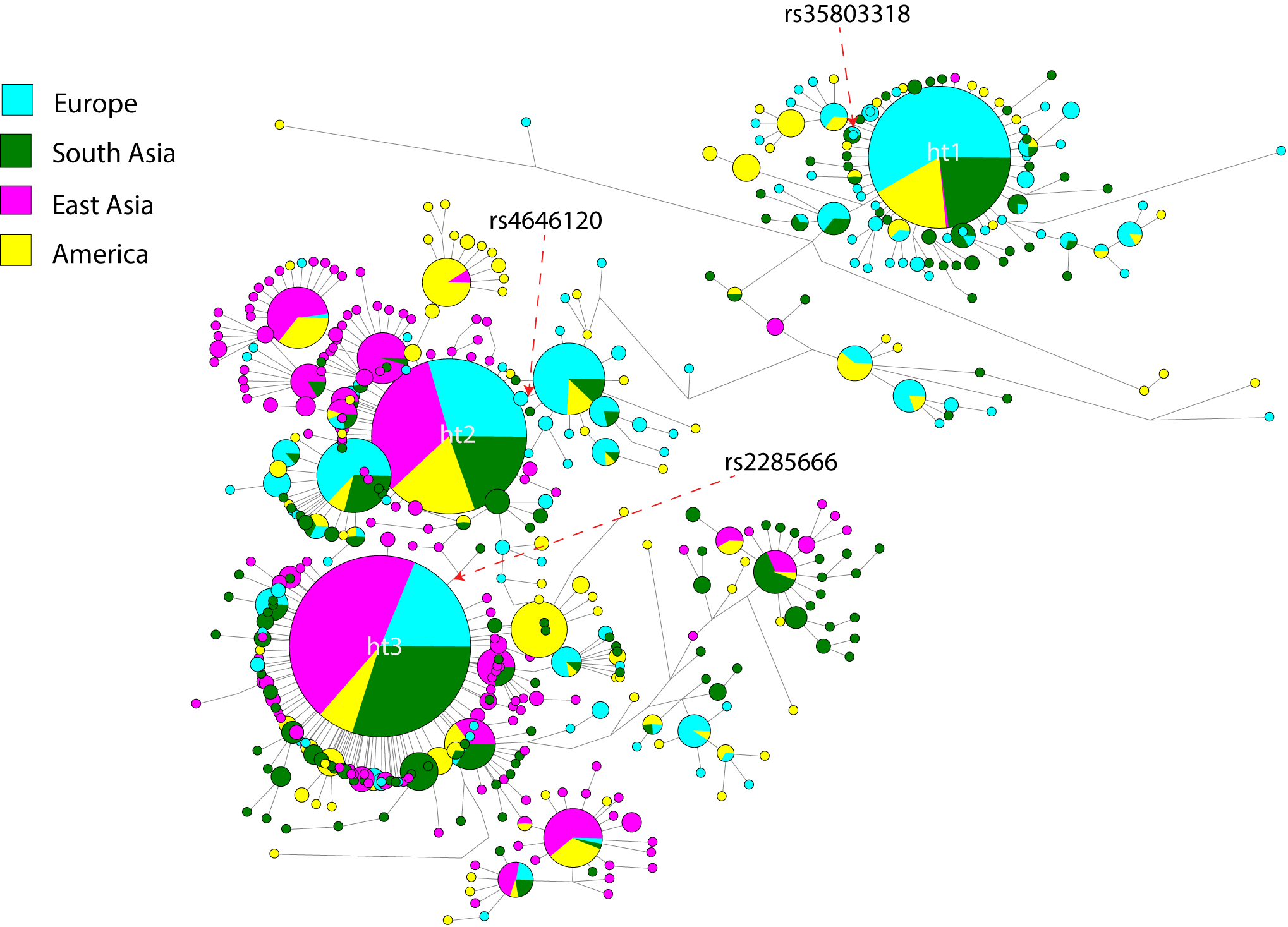

Supplement: S3 Fig — Circle sizes are proportional to the number of samples with that haplotype. The three most common haplotypes are marked. All three SNPs studied in detail have been marked by arrow. We used median joining method implemented in the NETWORK programme ver. 5. (TIF) [file pone.0238255.s003.tif]

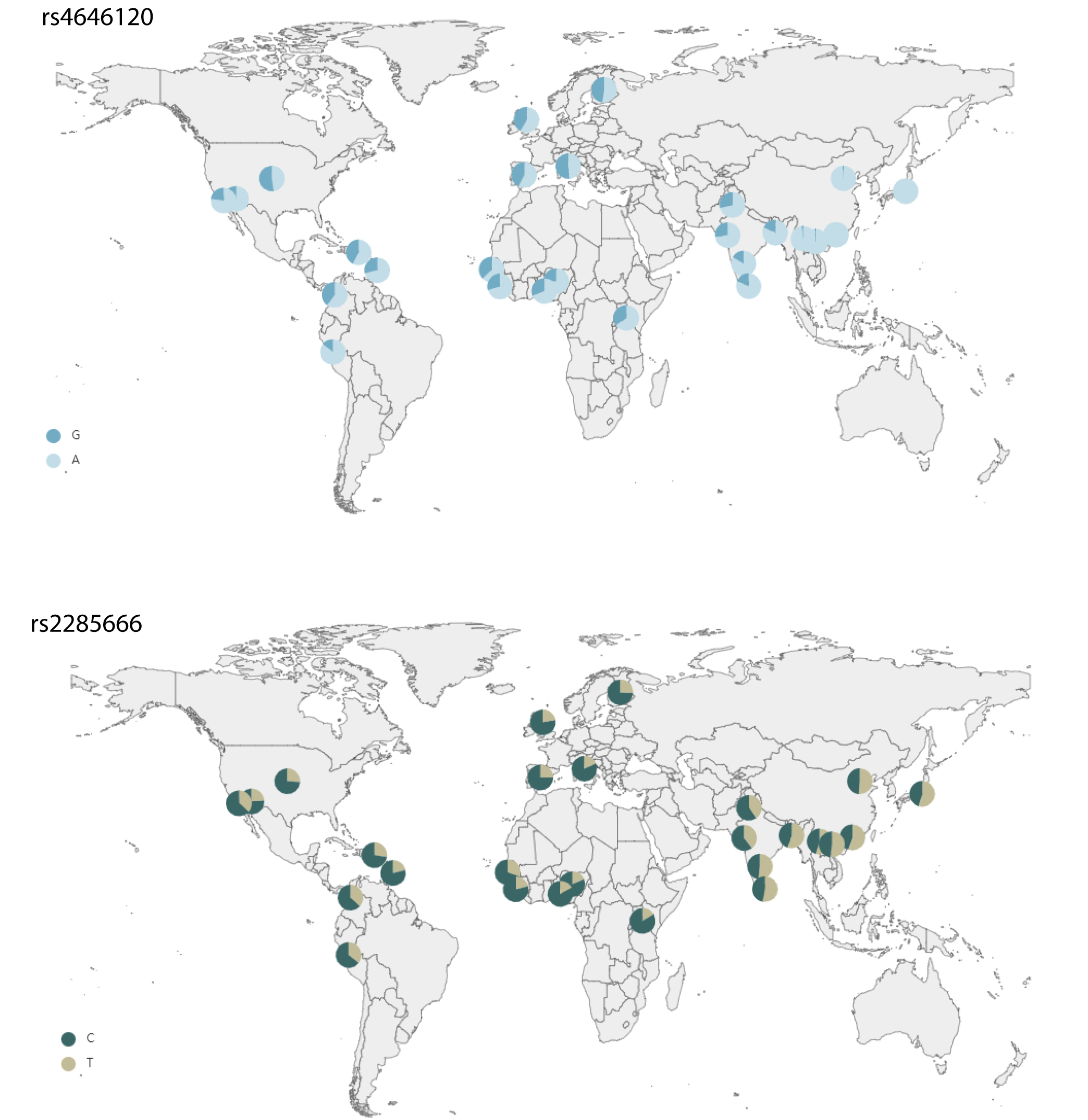

Supplement: S4 Fig — The map was obtained from the PGG toolkit implemented in the https://www.pggsnv.org/. (TIF) [file pone.0238255.s004.tif]
