# Supplementary material for: Most frequent South Asian haplotypes of ACE2 share identity by descent with East Eurasian populations
Source: PLoS One. 2020 Sep 16;15(9):e0238255. doi: 10.1371/journal.pone.0238255 (PMC7494073; doi:10.1371/journal.pone.0238255)
Supplement: S1 Table — The number of South Asian groups shown with their linguistic affiliations. (PDF) [file pone.0238255.s005.pdf]

Table S1: The geographic origin and number of samples analysed in the present study

Geographic Region			(N) Number of Samples
Europe			101
Caucasus			39
West Asia			20
Central Asia			24
South Asia			25
	Indo-European	18	
	Dravidian	4	
	Austroasiatic	3	
SEA_M (Southeast Asia_Mainland			18
SEA_I (Southeast Asia_Island			45
Siberia			108
America			13
