# Supplementary material for: Most frequent South Asian haplotypes of ACE2 share identity by descent with East Eurasian populations
Source: PLoS One. 2020 Sep 16;15(9):e0238255. doi: 10.1371/journal.pone.0238255 (PMC7494073; doi:10.1371/journal.pone.0238255)
Supplement: Attachment — Submitted filename: Reply to Reviewers.docx [file pone.0238255.s007.docx]

Reviewers' comments:

Reviewer's Responses to Questions

**Comments to the Author**

1. Is the manuscript technically sound, and do the data support the conclusions?

The manuscript must describe a technically sound piece of scientific research with data that supports the conclusions. Experiments must have been conducted rigorously, with appropriate controls, replication, and sample sizes. The conclusions must be drawn appropriately based on the data presented.

Reviewer #1: Partly

Reviewer #2: Yes

2. Has the statistical analysis been performed appropriately and rigorously?

Reviewer #1: N/A

Reviewer #2: Yes

3. Have the authors made all data underlying the findings in their manuscript fully available?

The [PLOS Data policy](http://www.plosone.org/static/policies.action#sharing) requires authors to make all data underlying the findings described in their manuscript fully available without restriction, with rare exception (please refer to the Data Availability Statement in the manuscript PDF file). The data should be provided as part of the manuscript or its supporting information, or deposited to a public repository. For example, in addition to summary statistics, the data points behind means, medians and variance measures should be available. If there are restrictions on publicly sharing data—e.g. participant privacy or use of data from a third party—those must be specified.

Reviewer #1: No

Reviewer #2: Yes

4. Is the manuscript presented in an intelligible fashion and written in standard English?

PLOS ONE does not copyedit accepted manuscripts, so the language in submitted articles must be clear, correct, and unambiguous. Any typographical or grammatical errors should be corrected at revision, so please note any specific errors here.

Reviewer #1: No

Reviewer #2: Yes

5. Review Comments to the Author

Please use the space provided to explain your answers to the questions above. You may also include additional comments for the author, including concerns about dual publication, research ethics, or publication ethics. (Please upload your review as an attachment if it exceeds 20,000 characters)

Reviewer #1: Srivastava et al present “Most frequent South Asian haplotypes of ACE2 share identity by descent with East Eurasian populations”. While the basic concept is interesting and timely given the current SARS-CoV-2 pandemic, the manuscript unfortunately can not be published in its current form:

**We have substantially revised the manuscript by addressing all the concerns of the reviewers.**

- Unfortunately important information is missing, reproducibility is not entirely given
- the manuscript was written quickly and includes several errors, which make it hard to read but also trust the results, e.g. wrong SNP name.
- Unfortunately the images in Figure1 where hardly readable, with the figure legends not very informative and helpful

**We have separated the figures now in the revised version.**

Major Issues:

The main issue I see with this manuscript is that it was written in a hustle, which a reader can easily tell, as there is information missing, one would typically expect, besides several errors (typos but also sentences, issues with references and figures). Comparison to 1000G data is very limited; as the main focus is on South Asia, with only 25 samples included, whereas 1000G includes 662 samples. Therefore one of my main concerns is, how representative are the 25 (mostly males, as ACE2 on X?) samples for South Asia. How do the results differ to the one reported by Cao et al 2020, what is the frequency of the two main SNPs in South-Asians for 1000G data?

**We have replicated our analysis with the 1000G data phase 3 data now in the revised version (Supplementary Figs 2-4). Results from both of the data has yielded similar results and conclusions drawn by us is supported by the analysis of independent datasets (Pagani et al. 2016 and 1000G).**

**We differ from Cao et al 2020 paper substantially. Cao et al have just looked the frequency of coding region substitutions among 1000G as well as Chinese dataset. They also didn’t add any analysis by the ancestry sharing point of view. Whereas, we used more robust haplotype based approach and moved a step ahead than Cao et al.**

**To have a better understanding of these SNPs worldwide, in the revised version, we have added the spatial distribution of two main SNPs in 1000G data (Supplementary Fig 4).**

The file in the provided data includes 402 samples, Pagani et al describe 483 samples. This work according the haplotypes in Suppl.Materials a total of 393 samples. Can you describe the discordance?

**According to the Supplementary Table 1. Pagani used 447 samples for their diversity panel. We have used the same data. After excluding regions e.g. Africa and Sahul as well as close relatives, we landed to 393 samples. We have explicitly mentioned this in the material and method section.**

Unfortunately (technical issue here) the figures can not be read well – can you please elaborate and include a larger font? Also the figure legends are not very informative.

**We have made separate figures now and also improved the legends.**

Please check the manuscript with help of a native english speaker

**We have included a co-author who has improved the quality of writing as well as edited the manuscript for the language.**

Minor Issues:

Abstract:
- include sample size of your data set, including for super populations once mentioned (EAS, SAS, EUR).
- the human Angiotensin-converting enzyme 2 (ACE2)
- more related TO West Eurasians
-focusing ON South Asia

**We have revised the manuscript as per the suggestions of the reviewer.**

-The second SNP – without talking about the first SNP? Why only the second one, what is with the first one?

**We have expanded the discussion about both of the SNPs in the main text.**

Introduction:
- Please recheck sentence: ...has been one of the serious threat to humanity now
- encoded by A gene located ON
- expression of ACE2 receptor? relate to the severity OF THE disease

**We have revised the sentences accordingly.**

- Confusing sentence: Growing evidence are accumulating, however, still the expression of ACE2 variations affecting host susceptibility among various world population is not known.

**We have rephrased the sentence.**

- South Asia is a country ...really? It is a region consisting of countries Afghanistan, Bangladesh, Bhutan, Maldives, Nepal, India, Pakistan and Sri Lanka

**We are sorry for this error! We have corrected it in the revised text.**

- Please recheck, very confusing: For the ACE2, it is not known that how the polymorphisms present in South Asia is shared with the East and West Eurasian populations?

**The sentence is revised now.**

-Reference 10 and 11 are identical, Reference 3 missing journal,

**We have rechecked all the references for the consistency.**

Material and Methods:
- Reference 10 vs 11. a [11] (Supplementary Table 1) here I was searching the data in Paganis Supplementary table – please make clearer

**These errors have been corrected.**

- Supplementary Figure 3: one can clearly see that the frequency of unique polymorphisms is strongly correletad to the sample size with one exception (SEA_M, this gets obvious when you order by sample size) – what is the purpose of the barplot? Same with Figure 1, how useful is this plot, can the sample size difference also be biasing the main finding?

**We agree with the reviewer and excluded these figures. To verify the main finding we used 1000G data as well as 26 random chromosomes for each of the groups. We didn’t get any deviation from the main finding in these analyses.**

- What is the purpose of supplemental table 1 and figure 3 – as figure 3 reports the same data?

**We agree with the reviewer. Since in the revised version we excluded Supp Fig. 3, we retained the Supplementary Table 1.**

- Please check sentence: … were constructed from the Haploview.

**It has been corrected now.**

ResultS and discussion

- Please check: Europeans and Siberians had THE highest number of private polymorphisms which ARE likely due to their large number of samples from these groups (Supplementary Figure 1, Figure 3, Table 1)

**We have excluded the figures as well as the sentence.**

- what are the snps defining ht1?

**We have updated Supp Table 1 SNPs with their location in different haplotypes.**

- LD patters → LD patterns

**It has been removed.**

- this unexpected result – wasn’t it reported by Cao et al 2020 yet?

**Cao et al. have looked the coding SNPs and didn’t analyse ancestry sharing, whereas we looked haplotypes and their sharing.**

- LD Plots – why are different amount of SNPs used for the subfigures on population level? However no chance to read. Can you please provide a better description of how the LD

**The program Haploview only consider the polymorphic SNPs for LD reconstruction, the polymorphic SNPs vary in different regions, therefore, the different amount of SNPs.**

- consistent naming of haplotypes ht1 … either with space or none

**It has been consistent now.**

- rs446120 is on chr8 – should be rs4646120 according figure 1c. 2X wrong in text.

**We are sorry for this typo, it is corrected in the manuscript.**

- was more common in West Eurasians

**Revised accordingly.**

- What leads to the conclusion that rs2285666 has lower severity for SARS-CoV-2?

**Recent studies have suggested that the Reference allele has reduced the ACE2 expression upto 50%, resulting the higher severity for SARS-CoV-2 (Singh et al. 2020; Wu et al., 2017; Asselta et al., 2020)**

Reviewer #2: Comments on the manuscript entitled “Most frequent South Asian haplotypes of ACE2 share identity by descent with East Eurasian populations”, by Srivastava et al.
Srivastava et al. have analysed whole genome data of the populations around the world, focusing South Asia, and extracted 248 ACE2 variations. Phylogenetic analysis of the above data suggested that the majority of South Asian ACE2 haplotypes are closer to the East Eurasians in the background of two unique event polymorphisms. Hence, the authors have suggested that it is highly likely that among the South Asians, the host susceptibility to the novel coronavirus SARS-CoV-2 will be more similar to East/Southeast Asians rather than the Europeans.

Considering the ongoing pandemic, this manuscript is appropriate now. However, I suggest the authors to take care of the following points.

**Thanks for the good words.**

1. No author has affiliation No. 5!

**We are sorry for this error. We have corrected it in the revised manuscript.**

2. No mention about the total number of samples, analysed; although supplementary Table 1 has the information. I suggest the authors to mention the numbers in the methods.

**We have revised the manuscript to reflect these informations.**

3. It may be useful to discuss in the manuscript about the linguistic background of South Asian populations that were included in the study.

**Thanks. We have added a supplementary table (table S1) with the linguistic information of South Asian samples.**

6. PLOS authors have the option to publish the peer review history of their article ([what does this mean?](https://journals.plos.org/plosone/s/editorial-and-peer-review-process#loc-peer-review-history)). If published, this will include your full peer review and any attached files.


If you choose “no”, your identity will remain anonymous but your review may still be made public.


**Do you want your identity to be public for this peer review?** For information about this choice, including consent withdrawal, please see our [Privacy Policy](https://www.plos.org/privacy-policy).

Reviewer #1: No

Reviewer #2: No


 **We hope that in the revised version you will find everything in order.**

**Sincerely,**

**G Chaubey (on behalf of coauthors)**
